# Predictors of COVID-19 vaccine hesitancy in Chad: A cross-sectional study

**DOI:** 10.3389/fpubh.2022.1063954

**Published:** 2023-01-04

**Authors:** Guy Rodrigue Takoudjou Dzomo, Edmond Mbario, Oumaima Djarma, Ndilbé Soumbatingar, Mouassede Madengar, Nadia Djimera, Allarangue Djindimadje, Christian Nguemadjita, Guirimadje Nassaringar, Margarita Bernales, Togoumbaye Nangerngar, Denise Naissem, Ephrem Paningar, Carlos Gomez-Virseda, Rodrigo Lopez Barreda, Ian Robbins, Amandine Cournil, Laurent Visier, Edouard Tuaillon, Franck J. D. Mennechet

**Affiliations:** ^1^University Hospital Complex “Le Bon Samaritain”, N'Djamena, Chad; ^2^Association for the Promotion of Village Health in Chad (APSVT), Bekamba, Chad; ^3^Republic of Chad, Ministry of Public Health and National Solidarity, N'Djamena, Chad; ^4^Faculty of Medicine, Pontificia Universidad Católica de Chile, Santiago, Chile; ^5^Center for Biomedical Ethics and Law, Department of Public Health and Primary Care, University KU Leuven, Leuven, Belgium; ^6^The Institute of Molecular Genetics of Montpellier (IGMM), University of Montpellier, CNRS, Montpellier, France; ^7^Institute of Research for Development (IRD), Montpellier, France; ^8^Center for Political and Social Studies (CEPEL), CNRS - University of Montpellier, Montpellier, France; ^9^Pathogenesis and Control of Chronic and Emerging Infections (PCCEI), INSERM U1058, University of Montpellier, French Blood Establishment (EFS), University of Antilles, Montpellier, France; ^10^Montpellier University Hospital (CHU), Montpellier, France

**Keywords:** vaccination, vaccine hesitancy, COVID-19, Sub-Saharan Africa, Chad

## Abstract

Vaccination against the COVID-19 virus is currently the best option to combat the SARS-CoV-2 pandemic worldwide. However, in addition to logistical and economic barriers, hesitancy to be vaccinated threatens to jeopardize efforts to contain the disease. An increasing number of people in Africa are delaying or rejecting recommended vaccines. Since their launch, COVID-19 vaccines have frequently faced rejection worldwide. In this study, we interviewed 5,174 participants from Chad that were representative of the general population, on their perception of COVID-19 vaccines. The survey was conducted from April to May 2021, before the rollout of the COVID-19 vaccination. We found that 47.9% of respondents were willing to receive the COVID-19 vaccine, 29.8% were undecided and 22.3% would not accept the vaccine. We found that urban residents were much more likely to refuse the vaccine than rural residents. We also observed that distrust of COVID-19 vaccines and mistaken beliefs played a crucial role in the reluctance to be vaccinated. Hesitancy to vaccinate against COVID-19 was strongly associated with lack of knowledge, and acceptance of vaccination was primarily associated with fear of the disease. Finally, we identified population profiles among the undecided and the refractors, which will help in developing strategies to combat COVID-19 vaccine resistance.

## Introduction

The emergence of the SARS-CoV-2 in December 2019, coupled with the lack of effective treatment has resulted in an unprecedented race for a vaccine. The control of this pandemic relies heavily on the acquisition of herd immunity, estimated at over 90% with the B.1.1.52 omicron variant (1). It took less than a year for the first emergency-use authorization of the COVID-19 vaccine to be issued on December 2020 by the U.S. Food and Drug Administration (2). As of August 2022, 170 vaccines against COVID-19 are in clinical development (3), and 38 have been approved by at least one country. Vaccines against COVID-19 have shown promising results to limit SARS-CoV-2 transmission and severe form of COVID-19, giving the world hope in the fight against this disease (4). To date, more than 12 billion doses of vaccine have been administered worldwide, representing 68% of the world's population having received at least one dose of a COVID-19 vaccine (5). However, there is great geographical variability, with only 22.7% of people in low-income countries having received at least one dose (5). This implies that vaccination efforts must still improve in low-income countries for the months and years to come.

While scientific efforts to develop the vaccine have been successful, the delivery of the vaccine has faced vast political, logistical, environmental and cultural barriers, especially in Africa (6). Hesitation to be vaccinated is one such obstacle. A part of each population refuses some vaccines (but accepts others), prefers to wait, or is unsure whether to be vaccinated. Vaccine hesitancy, has been defined by the Strategic Advisory Group of Experts on Immunization (SAGE) as “the delayed acceptance or refusal of vaccines despite the availability of immunization services” (7). According to the World health Organization (WHO), vaccines hesitancy represents a serious threat to global health, illustrated by the resurgence of some infectious diseases (8). Although it reflects individual factors, vaccine reluctance is complex in nature and is influenced by elements such as trust and convenience, fuelled by a mosaic of factors (9–11). Reluctance to COVID-19 vaccination is a worrying factor in the fight against the global pandemic, especially as the duration of protection of most COVID-19 vaccines remains uncertain and the emergence of new, more contagious, variants is a constant threat. Studies conducted around the world have shown that acceptance of the COVID-19 vaccine varies according to the period of the epidemic and the regions of the world (12, 13). There is significant geographical variability in vaccine hesitancy, ranging from 7 to 78% in high-income countries to 7 to 98% in low-income countries (9, 14, 15). Despite varying rates of vaccine hesitancy, about 30% of studies in Africa on COVID-19 vaccination reported higher than 50% hesitancy rate (14), and nearly half of the studies reported vaccine hesitancy of 30% or more in high-income countries (15). In general, the high levels of COVID-19 vaccine hesitancy recorded in many studies in Africa contrast with studies in other regions such as Europe and the Americas, China, Kuwait, and the UK (14, 16).

In Africa, the first COVID-19 vaccine doses of the continent arrived in Ghana on 24 February 2021 through the COVAX facility (17). Since then, 52 African countries have embarked on COVID-19 vaccination campaigns. However, Africa still lags far behind the rest of the world in vaccination coverage. As of October 4th, 2022, 163 doses of vaccine have been administered per 100 people worldwide, but only 46 and ~20 doses in Africa and Chad, respectively (5). While the international COVAX initiative aims to overcome the economic and logistical barriers to COVID-19 vaccine rollout in Africa, it is important to understand the way African people perceive these vaccines. It is also critical to identify the populations most distrustful of vaccines and the main reasons for this reluctance. Only then will it be possible to consider actions and develop the tools necessary to build acceptable trust in COVID-19 vaccines. While in December 2020, a survey showed that a majority (79%) of respondents in Africa would be willing to be vaccinated against COVID-19 if it was deemed safe and effective, further surveys conducted more recently have so far shown a disquieting level of COVID-19 vaccine hesitancy (14). Acceptance in the general population ranged from 15.4 to 33% in Cameroon to almost universal (97.9%) in Ethiopia (18–20).

For Chad (Box 1), no survey has ever been performed on vaccination hesitation or acceptance in general. The COVID-19 vaccination campaign started on 4 June 2021 with a donation of 200 000 doses of the Sinopharm^®^ vaccine from the Chinese government, and over 100 000 doses of the BioNtech Pfizer^®^ vaccine obtained through the COVAX/GAVI initiative. Between late 2021 and summer 2022, Chad received an additional 600,000 doses of the U.S. vaccine Ad26. COV2.s from Johnson & Johnson. Nevertheless, by the end of august 2022, < 15% of the population was fully vaccinated in Chad, making it one of the bottom 10 countries in the world in terms of COVID-19 vaccination coverage. Vaccination coverage in general has traditionally been low in this country. According to the WHO and UNICEF latest estimations, in 2021, BCG, Hepatitis B and poliomyelitis immunization coverage were respectively 75, 50, and 47% (21). The major aims of this study were therefore to determine the level of COVID-19 vaccine acceptance and hesitation in the general population in Chad (with the exception of healthcare workers), but also to identify predicting factors that might help to target the principal reasons and specific populations most prone to vaccine hesitancy.

Box 1 **Chad is a landlocked Sahelian country in Central Africa with a population of nearly 17 million, an area of 1,284,000 square kilometers, and borders with Sudan, the Central African Republic, Niger, Libya, and Nigeria.**

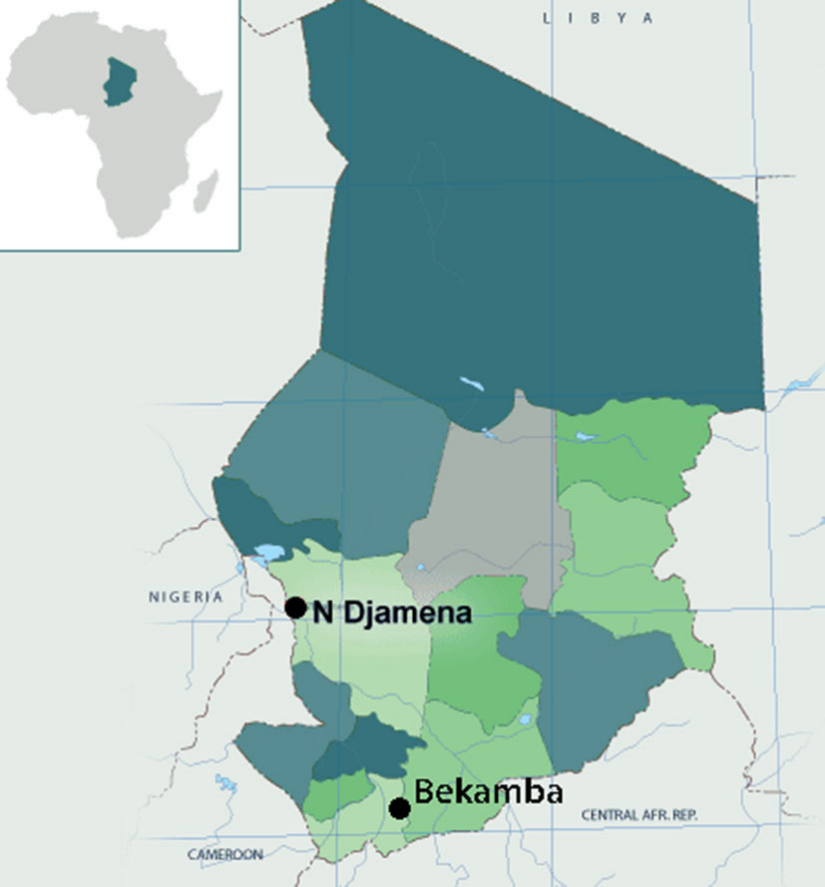

According to the United Nations Development Program, Chad ranks 182nd out of 184 countries on the Human Development Index (HDI: 0.398) in 2020.

In terms of religion, ~55% of the population is Muslim, 41% Christian and 1.4% traditional. Chad has been an oil-producing country since 2003, which now constitutes the basis of its economy, previously based on agriculture.

## Methods

### Study design and participants

A cross-sectional study was performed to collect data, using an anonymous administered questionnaire developed using the matrix of determinants of vaccine hesitancy generated by the WHO SAGE working group (7, 22). Adjustments were made to fit the specific context of Chad. This survey is a pilot study, conducted in the city of N'Djamena and in the sub-county of Bekamba. The interviewers used a random approach to meet with survey participants in different districts and areas of the cities of N'Djamena and Bekamba. We set the quotas according to the size of the two localities: about 5,000 for N'Djamena, which has nearly 1.7 million inhabitants, and about 1,000 for Bekamba and its surroundings, which have about 100,000 inhabitants. The investigators interviewed the participants directly and assisted them in a very neutral way when needed. The average time to complete a survey per participant was ~20 min. Investigators attended a 1-day training session to build consensus on methodology and optimal dissemination of the guidelines. An initial pilot survey was tested with 18 individuals and revised based on the feedback received, with minor modifications. Participants were asked if they were willing to participate in an anonymous survey on COVID-19 vaccination and informed that they could accept or decline without justification or consequences. Participants were also informed that the survey was free of charge, but also without financial compensation.

Criteria for eligibility in this study were being aged 18 years or older, with only one person per family questioned. To approach national representativeness, we purposely set quotas on gender, age, educational level and religion according to the data of the 4th survey of household conditions and poverty in Chad in 2018 (23). Persons under 18 years of age and the health service population (targeted for a separate study) were excluded from this survey. We contacted 6,850 people, of whom 960 refused to answer. The most frequent reason why persons refused to complete the survey was that they considered it requires too much effort whereas the temperatures exceeded regularly 40°C in April, Mai, corresponding to the warmer season in Ndjamena. Of the 5,890 questionnaires obtained, 716 were discarded, mainly because the central question on intention to be vaccinated was not answered.

The survey was fielded from 20 April to 14 May 2021, before the rollout of the COVID-19 vaccination in Chad. The study was approved by the institutional review board of the University Hospital Complex “Le Bon Samaritain” (IRB N°016/CHU-BS/DG/2021-B).

### Questionnaires

The questionnaire consisted of 36 questions divided into four sections. The participant first had to sign the following statement: “I certify that the nature and purpose of this study have been clearly explained to me and all my questions have been answered to my satisfaction. I agree to participate in this study voluntarily and without coercion.” The following parts were included in this questionnaire; (1) Questions addressing the socio-economic characteristics of the participants; (2) Questions evaluating participants' health status, both medical and past experiences with vaccination; (3) Items evaluating participants' knowledge and attitudes regarding COVID-19 and general perception of vaccination; (4) The final section measured the vaccination hesitancy variable and the reasons for different choices. All questions were close ended with either a single or multiple answer formats, including binary, yes/no scales, nominal and ordinal scales, and Likert type questions.

### Multivariate data analysis overview

The area-proportional Venn diagram method was used to compare three categories of determinants associated with COVID-19 vaccines hesitancy. Our classification does not follow a formal framework. The first category consisted of people “having a poor opinion of COVID-19 vaccination”; including participants who had had a bad experience in the past with vaccination, those who indicated that vaccines were often or always dangerous, or that vaccines never or rarely work. The second category included people who might be “less concerned about their vulnerability to COVID-19.” In this category, we integrated participants who reported being in excellent health, or who have had signs or a test for COVID-19 in the past year. The last category consisted of participants in “social withdrawal” that may be less concerned about spreading the SARS-CoV-2, including unemployed, single or housewife participants.

### Statistical analysis

The intention to be vaccinated against COVID-19 was measured by the following question: do you intend to be vaccinated against COVID-19 if the vaccine were available in Chad and free of charge? Reply options were: 1/ Yes, I will get vaccinated as soon as possible; 2/ I will observe for a while before deciding; 3/ It will depend on the type of vaccine; 4/ No, I will not get vaccinated; 5/ I don't know. In order to compare our study to other similar studies, answers were trichotomized for vaccination intention: willing (yes, I will get vaccinated as soon as possible), undecided (I will observe for a while before deciding, it will depend on the type of vaccine, and I don't know), unwilling (no, I will not get vaccinated). In this study, and in accordance with the SAGE working group, the term “hesitancy” includes undecideds as well as people who refuse COVID-19 vaccines. However, we have often separated people who are undecided to the one who rejected COVID-19 vaccination. We also sometimes use the term “reluctance” to vaccinate to refer to “vaccine hesitancy.”

Descriptive analyses were performed for all study variables. Bivariate analyses were run to test for association between each of the variables and the intention to be vaccinated. *P*-values for exact or Chi-2 tests were reported (Tables 1–3). A multivariable, logistic regression was used to assess the odds of undecidedness or unwillingness to receive COVID-19 vaccination using willingness as the reference category. Multivariable models were built for each set of predictors separately (sociodemographic, personal situation, beliefs and attitudes variables; Tables 4–6) and in each model all variables tested in biavariate analyses, were included. All *p*-values were two-sided, and statistical significance was set at *p* < 0.05. Statistical analyses were performed using the Stata software (version 15, Stata Corp., College Station, TX).

**Table 1:** Respondents' socio-demographic characteristics and association with COVID-19 vaccine intention.

**Characteristics**	**Respondents**	**COVID-19 vaccine intention**			***P*-value**
		**Yes**	**Undecided**	**No**	
	***n* (%)**	***n* (%)**	***n* (%)**	***n* (%)**	
**Total**	5,174	2,478 (47.9)	1,542 (29.8)	1,154 (22.3)	
Sex	5,019				0.34
Male	2,475 (49.3)	1,164 (47.0)	765 (30.9)	546 (22.1)	
Female	2,544 (50.7)	1,233 (48.5)	738 (29.0)	573 (22.5)	
**Age (years old)**	5,152				0.12
18–29	2,218 (43.1)	1,051 (47.4)	646 (29.1)	521 (23.5)	
30–39	1,497 (29.1)	718 (48.0)	437 (29.2)	342 (22.8)	
40–49	821 (15.9)	388 (47.3)	269 (32.8)	164 (20.0)	
50–59	429 (8.3)	207 (48.3)	131 (30.5)	91 (21.2)	
60 plus	187 (3.6)	104 (55.6)	53 (28.3)	30 (16.0)	
**Residence**	5,046				< 0.001
Urban	3,961 (78.5)	1,601 (40.4)	1,237 (32.7)	1,063 (26.8)	
Rural	1,085 (21.5)	824 (75.9)	210 (32.7)	51 (4.7)	
**Educational level**	4,972				0.001
No formal education	2,193 (44.1)	1,010 (46.1)	632 (28.8)	551 (25.1)	
Primary	785 (15.8)	368 (46.9)	243 (31.0)	174 (22.2)	
Secondary (ages: 11–18)	1,133 (22.8)	565 (49.9)	334 (29.5)	234 (20.7)	
University	861 (17.3)	393 (45.6)	300 (34.8)	168 (19.5)	
**Religion**	5,118				< 0.001
Muslim	2,502 (48.9)	1083 (43.3)	787 (31.5)	632 (25.3)	
Catholic	1,296 (25.3)	745 (57.5)	336 (25.9)	215 (16.6)	
Protestant	939 (18.3)	446 (47.5)	297 (31.6)	196 (20.9)	
Other	381 (7.4)	172 (45.1)	107 (28.1)	102 (26.8)	
**Marital status**	5,041				< 0.001
Married	2,685 (53.3)	1,414 (52.7)	727 (27.1)	544 (20.3)	
Single	1,368 (27.1)	582 (42.5)	449 (32.8)	337 (24.6)	
Widow	269 (5.3)	122 (45.4)	95 (35.3)	52 (19.3)	
Divorced	281 (5.6)	122 (43.4)	86 (30.6)	73 (26.0)	
Living with a partner	438 (8.7)	178 (40.6)	152 (34.7)	108 (24.7)	
**Occupation**	5,058				< 0.001
Student	1,032 (20.4)	522 (50.6)	303 (29.4)	207 (20.1)	
Manager	179 (3.5)	83 (46.4)	67 (37.4)	29 (16.2)	
Employee	386 (7.6)	193 (50.0)	103 (26.7)	90 (23.3)	
Trader	1,194 (23.6)	640 (53.6)	319 (26.7)	235 (19.7)	
Informal employment^*^	845 (16.7)	349 (41.3)	254 (30.1)	242 (28.6)	
Unemployed	1,036 (20.5)	479 (46.2)	311 (30.0)	246 (23.7)	
Housewife	386 (7.6)	152 (39.4)	155 (40.2)	79 (20.5)	

^*^Includes all occupations and forms of production carried out by people who receive an income but outside a legal framework.

**Table 2:** Respondents' personal situation and association with COVID-19 vaccine intention.

**Characteristics**	**Respondents**	**COVID-19 vaccine intention**			***P*-value**
		**Yes**	**Undecided**	**No**	
	***n* (%)**	***n* (%)**	***n* (%)**	***n* (%)**	
**Do you have a chronic disease?**	5,113				0.50
Yes	923 (18.1)	451 (48.9)	278 (30.1)	194 (21.0)	
No	4,190 (81.9)	19,91 (47.5)	1,244 (29.7)	955 (22.8)	
**How would you rate your current health status?**	4,970				0.002
Excellent	1,043 (21.0)	513 (49.2)	256 (24.5)	274 (26.3)	
Good	2,314 (46.6)	1,092 (47.2)	723 (31.2)	499 (21.6)	
Average	1,360 (27.4)	634 (46.6)	428 (31.5)	298 (21.9)	
Bad	221 (4.4)	118 (53.4)	63 (28.5)	40 (18.1)	
Very bad	32 (0.6)	14 (43.8)	10 (31.3)	8 (25.0)	
**Have you had any signs of COVID-19 in the last year?**	5,097				0.07
Yes	743 (14.6)	327 (44.0)	240 (32.3)	176 (23.7)	
No	4,354 (85.4)	2,113 (48.5)	1,277 (29.3)	964 (22.1)	
**Have you had a COVID-19 diagnostic test in the last year?**	5,139				< 0.001
Yes	530 (10.3)	324 (61.1)	139 (26.2)	67 (12.6)	
No	4,609 (89.7)	2,134 (46.3)	1,395 (30.3)	1,080 (23.4)	
**Have you ever had a friend or relative sick from COVID-19?**	5,161				0.003
Yes	740 (14.3)	357 (48.2)	250 (33.8)	133 (18.0)	
No	4,421 (85.7)	2,116 (47.9)	1,287 (29.1)	1,018 (23.0)	
**Have you ever had a friend or relative dead from COVID-19?**	5,144				0.20
Yes	463 (9.0)	222 (47.9)	151 (32.6)	90 (19.4)	
No	4,681 (91.0)	2,243 (47.9)	1,381 (29.5)	1,057 (22.6)	
**Are you afraid of contracting COVID-19?**	4879				< 0.001
Yes I am very afraid	1,749 (35.8)	1,092 (62.4)	418 (23.9)	239 (13.7)	
Yes I am a little afraid	1,176 (24.1)	621 (52.8)	361 (30.7)	194 (16.5)	
Yes sometimes	875 (17.9)	289 (33.0)	357 (40.8)	229 (26.2)	
Not at all	1,079 (22.1)	321 (29.7)	330 (30.6)	428 (39.7)	
**Have you had a medical examination at a hospital in the past year?**	5,138				0.005
Yes	1,735 (33.8)	854 (49.2)	539 (31.1)	342 (19.7)	
No	3,403 (66.2)	1,066 (47.2)	991 (29.1)	806 (23.7)	
**Have you had and international travel in the last 5 years**	5,119				0.12
Yes	430 (8.4)	213 (49.5)	138 (32.1)	79 (18.4)	
No	4,689 (91.6)	2,241 (47.8)	1,389 (29.6)	1,059 (22.6)	
**Do you have travel plans within the next 3 years**	5,140				< 0.001
Yes	949 (18.5)	401 (42.3)	357 (37.6)	191 (20.1)	
No	4,191 (81.5)	2,058 (49.1)	1,174 (28.0)	959 (22.9)	

**Table 3:** Respondent's beliefs/attitudes and association with COVID-19 vaccine intention.

**Characteristics**	**Respondents**	**COVID-19 vaccine intention**			***P*-value**
		**Yes**	**Undecided**	**No**	
	***n* (%)**	***n* (%)**	***n* (%)**	***n* (%)**	
**Vaccines protect against dangerous diseases**	5,005				< 0.001
Always	2,137 (42.7)	1,394 (65.2)	485 (22.7)	258 (12.1)	
Frequently	1,177 (23.5)	569 (48.3)	368 (31.3)	240 (20.4)	
Rarely	655 (13.1)	190 (29.0)	243 (37.1)	222 (33.9)	
Never	292 (5.8)	68 (23.3)	110 (37.7)	114 (39.0)	
I don't know	744 (14.9)	195 (26.2)	283 (38.0)	266 (35.8)	
**Have you ever hesitated to vaccinate yourself or a relative in the past?**	5,064				< 0.001
Always	409 (8.1)	147 (35.9)	131 (32.0)	131 (32.0)	
Frequently	565 (11.2)	161 (28.5)	227 (40.2)	177 (31.3)	
Rarely	919 (18.1)	394 (42.9)	305 (33.2)	220 (23.9)	
Never	2,679 (52.9)	1,543 (57.6)	683 (25.5)	453 (16.9)	
I don't know	492 (9.7)	169 (34.3)	170 (34.6)	153 (31.1)	
**Have you ever refused a vaccine for yourself or a relative in the past?**	5,127				< 0.001
Yes	647 (12.6)	119 (18.4)	219 (33.8)	309 (47.8)	
No	4,418 (86.2)	2,316 (52.4)	1,282 (29.0	820 (18.6)	
I don't know	62 (1.2)	15 (24.2)	31 (50.2)	16 (25.8)	
**Have you ever had a negative experience with vaccination in the past?**	5,056				< 0.001
Yes	905 (17.9)	229 (25.3)	314 (34.7)	362 (40.0)	
No	3,747 (74.1)	2,028 (54.1)	1,063 (28.4)	656 (17.5)	
I don't know	404 (8.0)	163 (40.3)	136 (33.7)	105 (26.0)	
**Have you ever been prevented from getting a vaccination because of distance, lack of time or money?**	5,060				< 0.001
Yes	1,558 (30.8)	661 (42.4)	536 (34.4)	361 (23.2)	
No	3,275 (64.7)	1,647 (50.3)	918 (28.0)	710 (21.7)	
I don't know	227 (4.5)	90 (39.6)	71 (31.3)	66 (29.1)	
**Have you ever received negative information on vaccination from a relative?**	5,069				< 0.001
Yes	1,748 (34.5)	585 (33.5)	607 (34.7)	556 (31.8)	
No	3,109 (61.3)	1,754 (56.4)	831 (26.7)	524 (16.9)	
I don't know	212 (4.2)	83 (39.2)	81 (38.2)	48 (22.6)	
**Have you ever received negative information on vaccination from mainstream media?**	5,142				< 0.001
Yes	1,005 (19.5)	342 (34.0)	363 (29.9)	300 (29.9)	
No	3,800 (73.9)	2,020 (53.2)	1,036 (27.3)	744 (19.6)	
I don't know	337 (6.6)	99 (29.4)	131 (38.9)	107 (31.8)	
**Have you ever received negative information on vaccination from social media?**	5,139				< 0.001
Yes	1,082 (21.1)	387 (35.8)	410 (37.9)	285 (26.3)	
No	3,698 (72.0)	1,948 (52.7)	998 (27.0)	752 (20.3)	
I don't know	359 (7.0)	123 (34.3)	125 (34.8)	111 (30.9)	
**Vaccines are dangerous or cause diseases**	5,049				< 0.001
Always	434 (8.6)	129 (29.7)	118 (27.2)	187 (43.1)	
Frequently	642 (12.7)	176 (27.4)	229 (35.7)	237 (36.9)	
Rarely	985 (19.5)	427 (43.4)	348 (35.3)	210 (21.3)	
Never	1,782 (35.3)	1,207 (67.7)	388 (21.8)	187 (10.5)	
I don't know	1,206 (23.9)	463 (38.4)	433 (35.9)	310 (25.7)	
**African traditions are against vaccination**	5,098				< 0.001
Yes	1,174 (23.0)	423 (36.0)	407 (34.7)	344 (29.3)	
No	3025 (59.3)	1726 (57.1)	789 (26.1)	510 (16.9)	
I don't know	899 (17.6)	288 (32.0)	326 (36.3)	285 (31.7)	
**Religion and faith are against vaccination**	5,096				< 0.001
Yes	619 (12.1)	190 (30.7)	226 (36.5)	203 (32.8)	
No	3,651 (71.6)	1,995 (54.6)	984 (27.0)	672 (18.4)	
I don't know	826 (16.2)	253 (30.6)	316 (38.3)	257 (31.1)	
**Knowing leaders in Chad who are against vaccination**	4,991				< 0.001
Yes	576 (11.5)	207 (35.9)	203 (35.2)	166 (28.8)	
No	3,688 (73.9)	1,906 (51.7)	1,039 (28.2)	743 (20.1)	
I don't know	727 (14.6)	265 (36.5)	254 (34.9)	208 (28.6)	
**Vaccination is a tool used to sterilize black people**	5,077				< 0.001
Yes	791 (15.6)	166 (21.0)	273 (34.5)	352 (44.5)	
No	3,160 (62.2)	1,846 (58.4)	849 (26.9)	465 (14.7)	
I don't know	1,126 (22.2)	417 (37.0)	397 (34.9)	316 (28.1)	
**Vaccination is a way to introduce diseases in Africa**	5,053				< 0.001
Yes	1,295 (25.6)	261 (20.2)	451 (34.8)	583 (45.0)	
No	2,676 (53.0)	1,725 (64.5)	665 (24.9)	286 (10.7)	
I don't know	1,082 (21.4)	428 (39.6)	385 (35.6)	269 (24.9)	
**Vaccination is a mean from the West to get more money**	5,117				< 0.001
Yes	1,466 (28.6)	409 (27.9)	501 (34.2)	556 (37.9)	
No	2,379 (46.5)	1,550 (65.2)	559 (23.5)	270 (11.3)	
I don't know	1,272 (24.9)	490 (38.5)	464 (36.5)	318 (25.0)	

**Table 4:** Socio-demographic predictors of COVID-19 vaccine undecidedness or rejection: multivariate analysis.

**Characteristics**	**COVID-19 vaccine undecidedness**				**COVID-19 vaccine rejection**			
	**aOR**	**95% CI**		***P*-value**	**aOR**	**95% CI**		***P*-value**
Male	Ref				Ref			
Female	0.88	0.75	1.04	0.130	0.86	0.71	1.03	0.102
**Age (years old)**								
18–29	Ref				Ref			
30–39	1.05	0.86	1.28	0.617	0.93	0.75	1.16	0.540
40–49	1.42	1.12	1.81	0.004	0.88	0.67	1.16	0.378
50–59	1.32	0.98	1.78	0.068	0.97	0.68	1.38	0.864
60 plus	1.02	0.67	1.55	0.934	0.66	0.39	1.10	0.110
**Residence**								
Urban	Ref				Ref			
Rural	0.39	0.32	0.48	< 0.001	0.10	0.07	0.13	< 0.001
**Educational level**								
No formal education	Ref				Ref			
Primary	1.12	0.91	1.38	0.287	0.86	0.68	1.10	0.228
Secondary	1.10	0.88	1.37	0.412	0.72	0.55	0.93	0.012
University	1.21	0.93	1.56	0.154	0.61	0.45	0.82	0.001
**Religion**								
Muslim	Ref				Ref			
Catholic	0.88	0.73	1.06	0.189	0.97	0.78	1.21	0.796
Protestant	1.02	0.83	1.24	0.860	1.15	0.91	1.44	0.247
Other	0.85	0.63	1.13	0.259	1.21	0.89	1.64	0.234
**Marital status**								
Married	Ref				Ref			
Single	1.52	1.23	1.87	< 0.001	1.31	1.04	1.66	0.022
Widow	0.95	0.68	1.33	0.785	1.12	0.78	1.61	0.533
Divorced	1.11	0.82	1.50	0.506	0.74	0.51	1.08	0.117
Living with a partner	1.42	1.09	1.85	0.008	1.12	0.84	1.51	0.440
**Occupation**								
Student	Ref				Ref			
Manager	1.27	0.83	1.93	0.266	1.10	0.66	1.83	0.729
Employee	0.97	0.70	1.35	0.865	1.15	0.79	1.67	0.460
Trader	1.22	0.92	1.61	0.167	1.25	0.90	1.74	0.176
Informal employment	1.41	1.06	1.86	0.017	1.58	1.15	2.17	0.005
Enemployed	1.89	1.38	2.59	< 0.001	1.33	0.90	1.96	0.151
Housewife	1.58	1.17	2.12	0.003	1.54	1.10	2.15	0.013

**Table 5:** Personal situations predictors of COVID-19 vaccine undecidedness or rejection: multivariate analysis.

**Characteristics**	**COVID-19 vaccine undecidedness**				**COVID-19 vaccine rejection**			
	**aOR**	**95% CI**		***P*-value**	**aOR**	**95% CI**		***P*-value**
**Having a chronic disease**								
Yes	1.03	0.84	1.26	0.788	1.17	0.92	1.48	0.190
No	Ref				Ref			
**Current health status estimation**								
Excellent	Ref				Ref			
Good	1.34	1.10	1.63	0.003	0.96	0.78	1.18	0.709
Average	1.36	1.09	1.70	0.006	0.98	0.77	1.25	0.874
Bad	1.18	0.80	1.74	0.401	0.75	0.47	1.18	0.216
Very bad	1.22	0.50	2.97	0.667	1.51	0.56	4.04	0.417
**Having COVID-19 signs since one year**								
Yes	1.23	0.99	1.52	0.058	1.60	1.26	2.04	< 0.001
No	Ref				Ref			
**Having COVID-19 diagnostic test**								
Yes	0.64	0.50	0.81	< 0.001	0.53	0.38	0.72	< 0.001
No	Ref				Ref			
**Having a friend or relative sick from COVID-19**								
Yes	1.19	0.94	1.50	0.156	0.82	0.61	1.11	0.202
No	Ref				Ref			
**Having a friend or relative dead from COVID-19**								
Yes	1.12	0.85	1.49	0.419	1.25	0.87	1.80	0.220
No	Ref				Ref			
**Fear of getting COVID-19**								
Yes I am very afraid	0.35	0.29	0.44	< 0.001	0.16	0.13	0.20	< 0.001
Yes I am a little afraid	0.57	0.46	0.70	< 0.001	0.23	0.18	0.28	< 0.001
Yes sometimes	1.15	0.91	1.44	0.246	0.55	0.43	0.69	< 0.001
Not at all	Ref				Ref			
**Having medical visit in a hospital since one year**								
Yes	Ref				Ref			
No	1.02	0.87	1.19	0.838	0.86	0.71	1.04	0.114
**Having international travel in the last 5 years**								
Yes	0.89	0.68	1.16	0.383	0.80	0.57	1.11	0.185
No	Ref				Ref			
**Having travel plans within the next 3 years**								
Yes	1.60	1.33	1.93	< 0.001	1.00	0.80	1.26	0.976
No	Ref				Ref			

**Table 6:** Beliefs and attitudes predictors of COVID-19 vaccine undecidedness or rejection: multivariate analysis.

**Characteristics**	**COVID-19 vaccine undecidedness**				**COVID-19 vaccine rejection**			
	**aOR**	**95% CI**		***P*-value**	**aOR**	**95% CI**		***P*-value**
**Vaccines protect against dangerous diseases**								
Always	Ref				Ref			
Frequently	1.34	1.07	1.67	0.011	1.75	1.32	2.32	< 0.001
Rarely	1.81	1.36	2.40	< 0.001	2.74	1.96	3.83	< 0.001
Never	2.50	1.68	3.73	< 0.001	4.28	2.71	6.77	< 0.001
I don't know	1.99	1.48	2.68	< 0.001	2.94	2.09	4.12	< 0.001
**Parents in Chad get their children vaccinated at birth**								
Always	Ref				Ref			
Frequently	1.06	0.85	1.33	0.612	0.97	0.73	1.29	0.829
Rarely	1.17	0.92	1.48	0.204	1.46	1.10	1.95	0.010
Never	1.83	1.23	2.74	0.003	1.30	0.77	2.20	0.326
I don't know	1.46	1.04	2.05	0.028	0.93	0.62	1.40	0.729
**Hesitation to get vaccinated or to have a relative vaccinated in the past**								
Always	1.45	1.03	2.04	0.033	1.37	0.90	2.07	0.141
Frequently	2.12	1.59	2.84	< 0.001	1.31	0.91	1.90	0.149
Rarely	1.24	0.99	1.54	0.058	0.92	0.70	1.22	0.565
Never	Ref				Ref			
I don't know	1.32	0.96	1.81	0.085	1.89	1.32	2.70	0.001
**Rejection of a vaccine for oneself or a relative in the past**								
No	Ref				Ref			
Yes	1.47	1.06	2.04	0.021	2.46	1.73	3.48	< 0.001
I don't know	1.49	0.60	3.72	0.395	1.08	0.34	3.41	0.895
**Negative experience with vaccination in the past**								
No	Ref				Ref			
Yes	1.31	1.03	1.68	0.030	1.84	1.38	2.44	< 0.001
I don't know	0.69	0.49	0.98	0.037	0.70	0.47	1.04	0.080
**Prevented from distance. time or money to get a vaccine in the past**								
No	Ref				Ref			
Yes	1.03	0.86	1.24	0.754	0.82	0.65	1.03	0.087
I don't know	0.92	0.60	1.42	0.713	0.99	0.60	1.62	0.966
**Having received negative information on vaccination from a relative**								
No	Ref				Ref			
Yes	1.12	0.93	1.36	0.237	1.09	0.86	1.38	0.496
I don't know	1.16	0.71	1.88	0.556	0.88	0.49	1.59	0.676
**Having received negative information on vaccination from mainstream media**								
No	Ref				Ref			
Yes	1.19	0.94	1.51	0.157	1.06	0.79	1.43	0.708
I don't know	1.48	0.97	2.24	0.067	1.13	0.70	1.84	0.611
**Having received negative information on vaccination from social media**								
No	Ref				Ref			
Yes	1.25	0.99	1.57	0.060	1.05	0.79	1.41	0.722
I don't know	1.08	0.74	1.58	0.681	1.36	0.88	2.09	0.163
**Vaccines are dangerous or cause diseases**								
Always	1.19	0.82	1.73	0.366	2.56	1.70	3.86	< 0.001
Frequently	1.83	1.36	2.47	< 0.001	2.26	1.58	3.23	< 0.001
Rarely	1.62	1.29	2.04	< 0.001	1.71	1.26	2.31	0.001
Never	Ref				Ref			
I don't know	1.38	1.08	1.76	0.010	1.82	1.35	2.46	< 0.001
**African traditions are against vaccination**								
No	Ref				Ref			
Yes	1.12	0.89	1.40	0.345	1.02	0.77	1.35	0.883
I don't know	1.13	0.85	1.51	0.389	1.48	1.06	2.07	0.020
**Religion and faith are against vaccination**								
No	Ref				Ref			
Yes	1.14	0.84	1.54	0.405	1.32	0.92	1.90	0.132
I don't know	1.53	1.14	2.05	0.004	1.25	0.88	1.77	0.214
**Knowing leaders in the Republic of Chad who are against vaccination**								
No	Ref				Ref			
Yes	0.84	0.63	1.13	0.255	0.72	0.50	1.04	0.079
I don't know	0.93	0.71	1.21	0.588	0.93	0.69	1.26	0.660
**Vaccination is a tool used to sterilize black people**								
No	Ref				Ref			
Yes	1.06	0.77	1.45	0.725	1.15	0.82	1.62	0.421
I don't know	0.93	0.71	1.21	0.577	0.98	0.71	1.34	0.889
**Vaccination is a way to introduce diseases in Africa**								
No	Ref				Ref			
Yes	1.77	1.36	2.32	< 0.001	3.97	2.93	5.39	< 0.001
I don't know	1.03	0.79	1.36	0.811	1.44	1.04	2.01	0.029
**Vaccination is a mean from the West to get more money**								
No	Ref				Ref			
Yes	1.83	1.46	2.30	< 0.001	2.41	1.83	3.18	< 0.001
I don't know	1.45	1.15	1.84	0.002	1.66	1.23	2.23	0.001

## Results

### Characteristics of the respondents

Of the 6,850 people who were asked to participate in the questionnaire, ~14% refused and ~10% were rejected due to an invalid questionnaire. Valid responses were received from 5,174 respondents, representing ~76% of the people approached for this study.

### Socio-demographic characteristics

The socio-demographic characteristics are shown in Table 1. The mean age of the respondents was 33.5 years (SD: 11.9 years). There was a roughly equal proportion of female respondents (50.7%) to male (49.3%). Most respondents were aged 18–39 years (72.2%). The majority of the respondents (78.5%) lived in the urban area of N'Djamena, while 21.5% lived in rural areas. About 44 % of the respondents had no formal education and 15.8% only attended primary school. Almost half (48.9%) of the participants were Muslim, 25.3% Catholic and 18.3% Protestant. Married people represented 53.3% of the respondents and the most represented by occupation were shopkeepers (traders) (23.6%), unemployed (20.5%), students (20.4%) and people with informal employment (16.7%).

### Personal situation characteristics

The personal characteristics of the participants are presented in Table 2. Most of the participants (67.6%) reported to be in excellent or good health while 18% had an underlying chronic disease. Approximately 15% of participants had noticed signs of COVID-19 in the past year, and ~23% reported having a friend or relative who was ill or died from COVID-19. The fear of getting COVID-19 was noted at various degrees: very afraid (35.8%), little afraid (24.1%), sometimes afraid (17.9%) and not afraid at all (22.1%). About one third of the respondents had a medical visit in a hospital during the last year (33.8%), and 18.5% had international travel plans within the next 3 years.

### Beliefs and attitudes characteristics

The characteristics of the respondents in terms of beliefs and attitudes are presented in Table 3. Briefly, most respondents believe vaccines always protect against dangerous diseases (42.7%) or frequently (23.5%). About half of the participants (52.9%) had never hesitated to get vaccinated or to have a relative vaccinated in the past, while 86.2% had never rejected a vaccine for themselves or for a relative. The majority of the participant (74.1%) had no negative experience with vaccines. With regards to the circulation of negative information on vaccination, 34.5% of the respondents acknowledged the reception of negative information from a relative and 21.1% from social media. In addition, ~21% of the participants believed that vaccines are always, or often dangerous and 34% indicated that African traditions or religion are against vaccination. Finally, 15.6% of the participants indicated that vaccination is a tool used to sterilize black people, 25.6% a way to introduce diseases into Africa, and 28.6% a way for the West to enrich itself.

### Intention to be vaccinated against COVID-19 and perception of the risks of the disease

The overall vaccine intention rate in our study population was as follows: 47.9% of the respondents were willing to get a COVID-19 vaccines if it were available free of charge in Chad, 29.8% were undecided and 22.3% would not accept the vaccine (Table 1). These rates were similar across genders. For the other socio-demographic characteristics, acceptance of the COVID-19 vaccine was significantly higher among respondents living in rural areas (75.9%). The rate of undecidedness was highest among housewives (40.2%). The rejection rate of COVID-19 vaccine was highest in participants with informal employment (28.6%). With regard to characteristics on personal situations (Table 2), the acceptance rate was highest in respondents who were very afraid of getting COVID-19 (62.4%). An inverse pattern was observed with the rates of undecidedness and rejection. With respect to the characteristics on beliefs and attitudes (Table 3), acceptance of the COVID-19 vaccine was highest among respondents who never believed that vaccines were dangerous or cause diseases (67.7%). In contrast, COVID-19 vaccine acceptance rate was lower in people who had rejected a vaccine for themselves or for a relative in the past (18.4%), or for those who believed that vaccination is a tool used to sterilize black people (21.0%), or to introduce diseases in Africa (20.2%). An inverse pattern was observed with the rates of undecidedness and rejection.

### Main reasons for acceptance or hesitancy of COVID-19 vaccination

**Figure 1:**
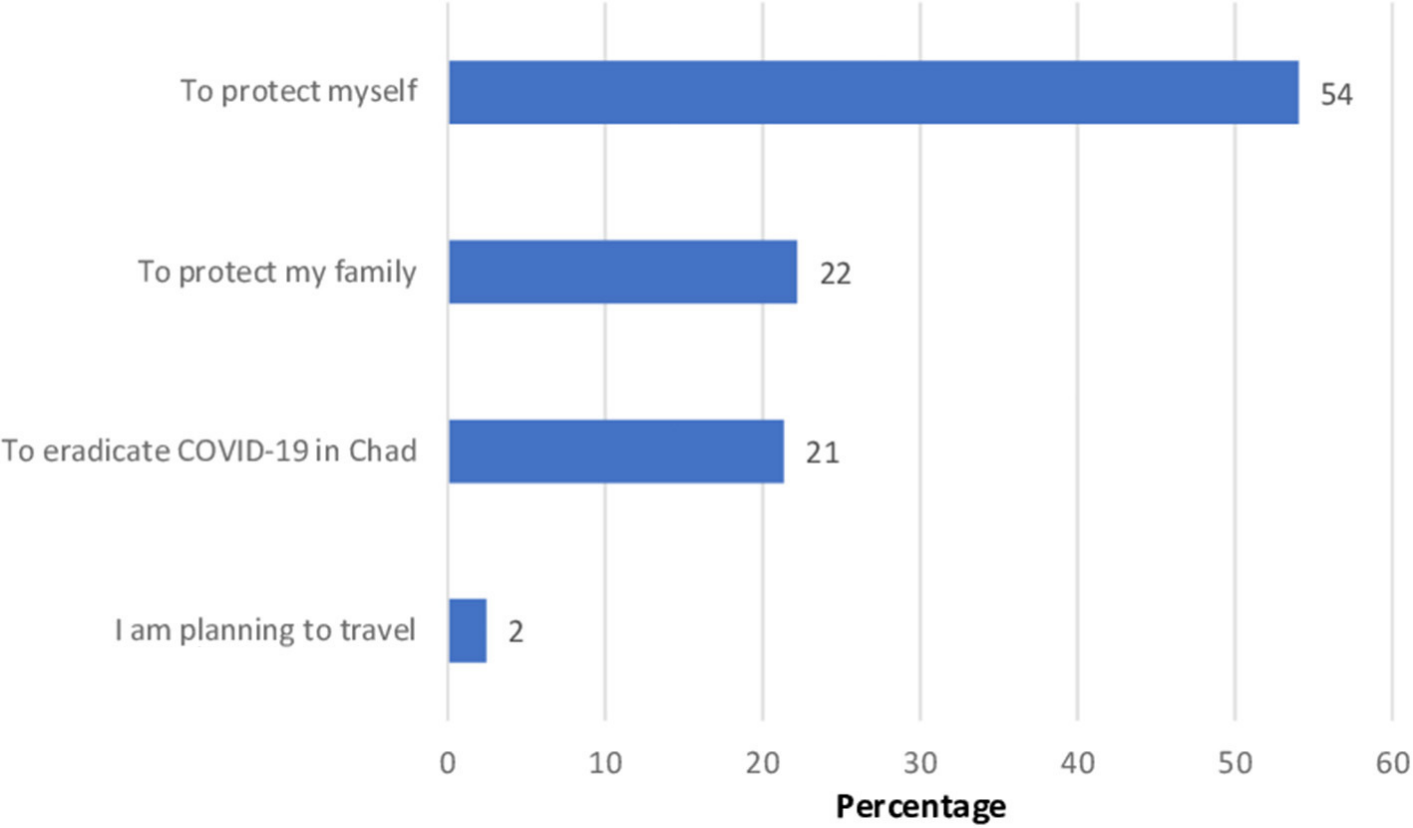
Listed reasons to be vaccinated against COVID-19, ranked in order of importance (in percent) among participants who are willing to be vaccinated (*n* = 2,477).

**Figure 2:**
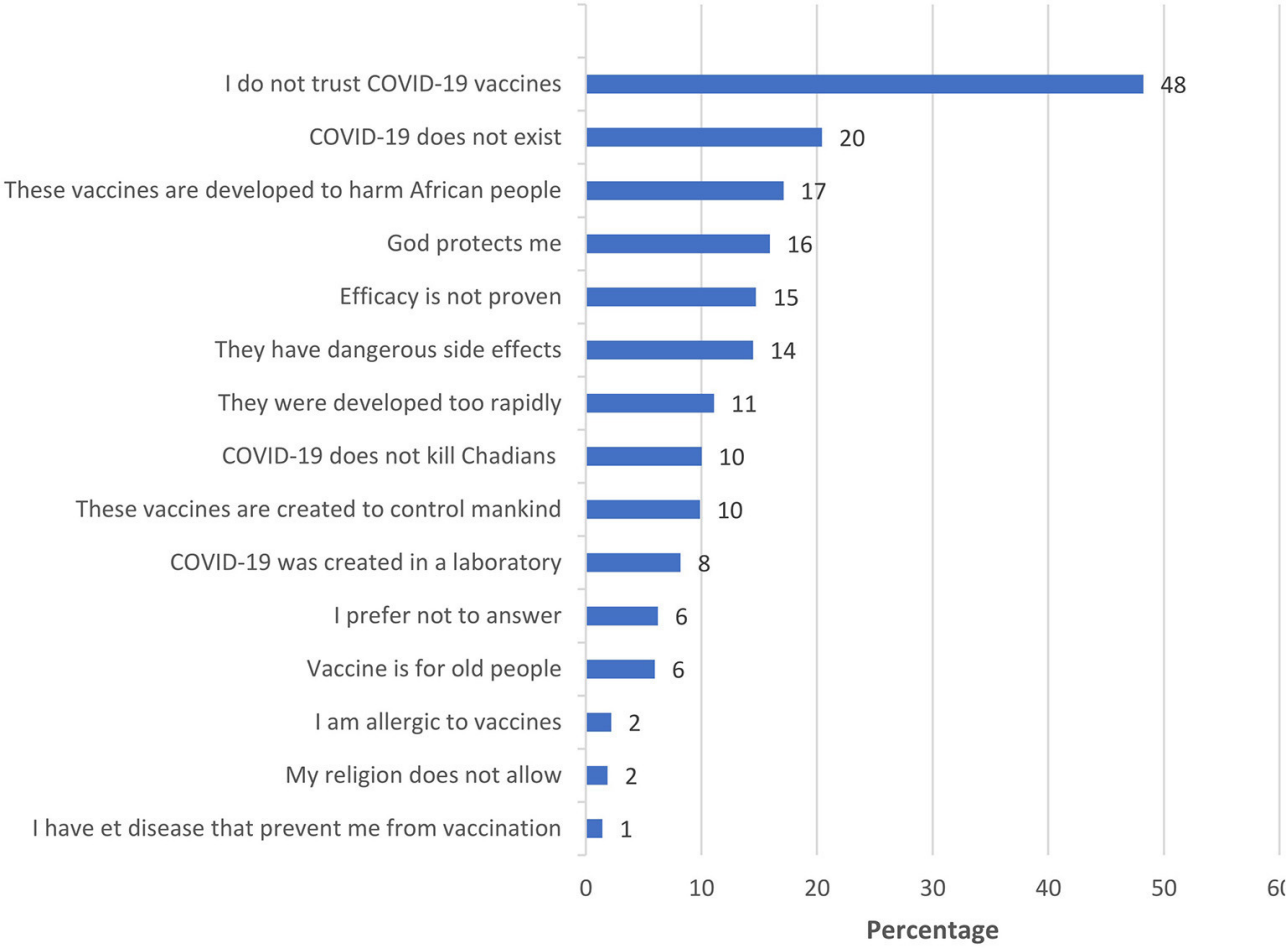
Listed reasons for reluctance to be vaccinated against COVID-19, ranked in order of importance (in percent) among undecided or vaccine-refractory participants (*n* = 2,697).

The reasons for acceptance of the COVID-19 vaccination by the participants are listed and ranked in Figure 1. The main reasons given by the respondents were to protect themselves (54%), or their family (22%), to eradicate COVID-19 in Chad (21%), and finally, 2% of the respondents intended to accept the COVID-19 vaccine because they plan to travel. For those who were either undecided or rejected COVID-19 vaccine, reasons of hesitancy are listed and ranked in Figure 2. Main reasons were lack of confidence in COVID-19 vaccines (48%), followed by 20% believing that COVID-19 did not exist, 17% believing that COVID-19 vaccines were being developed to harm Africans, 16% relying on God for protection, 15% believing that the effectiveness of the vaccine is not proven, and 14% indicated that vaccines have dangerous side effects.

### Multivariate logistic regression analysis

Results of the multivariate logistic regression analysis of the predictors of COVID-19 vaccine undecidedness and rejection are shown with respect to socio-demographic characteristics (Table 4), personal characteristics (Table 5), and characteristics related to beliefs and attitudes (Table 6).

### Socio-demographic predictors of COVID-19 vaccine undecidedness or rejection

With regard to undecidedness on COVID-19 vaccine intention, 17 characteristics were found to be significantly associated (Table 4). In the socio-demographic category, high significance (*p* < 0.001) was found for participants living in rural areas compared to those living in urban areas (aOR: 0.39; 95% CI: 0.32–0.48); respondents living with a partner compared to married respondents (aOR: 1.42; 95% CI: 1.09–1.85); and unemployed compared to students (aOR: 1.89; 95% CI: 1.38–2.59).

Concerning COVID-19 vaccine rejection, 14 characteristics were significantly associated. The strongest associations were also found between participants living in rural areas compared to urban respondents (aOR: 0.10; 95% CI: 0.07–0.13).

### Personal situations predictors of COVID-19 vaccine undecidedness or rejection

In the category of personal situation characteristics (Table 5), predictors of undecidedness about the COVID-19 vaccine were strongest between respondents who had undergone COVID-19 diagnostic testing and those who had not (aOR: 0.64; 95% CI: 0.50–0.81); respondents who were very afraid of getting COVID-19 as compared to those with no fear of getting COVID-19 (aOR: 0.35; 95% CI: 0.29–0.44); respondents with travel plans within the next 3 years as compared to those with no travel plans (aOR: 1.60:; 95% CI: 1.33–1.93).

The following characteristics were most strongly associated with rejection of the COVID-19 vaccine: respondents who had had COVID-19 signs during the past year as compared to those who had not (aOR: 1.60: 95% CI: 1.26–2.04); respondents who had had a COVID-19 diagnostic test as compared to those who did not test (aOR: 0.53; 95% CI: 0.38–0.72); and finally, respondents who were very afraid of getting COVID-19, as compared to those with no fear (aOR: 0.16; 95% CI: 0.13–0.20).

### Beliefs and attitudes predictors of COVID-19 vaccine undecidedness or rejection

Within the category of beliefs and attitudes (Table 6), the following characteristics were most strongly associated with undecidedness about the COVID-19 vaccine: participants who believed that vaccines rarely protected against dangerous diseases (aOR: 1.81; 95% CI: 1.36–2.40) or never (aOR: 2.50; 95% CI: 1.68–3.73) compared to those who indicated that vaccines always protected against sever diseases; respondents who have frequently hesitated to get vaccinated or to have a relative vaccinated in the past as compared to those who did not (aOR: 2.12; 95% CI: 1.59–2.84); respondents who believed vaccines are dangerous or cause diseases, either frequently or rarely as compared to those who did not (aOR: 1.83; 95% CI: 1.36–2.47 and aOR: 1.62: 95% CI: 1.29–2.04, respectively); respondents who believed that vaccination was a way to introduce diseases into Africa as compared to those who do not believe so (aOR: 1.77; 95% CI: 1.36–2.32); and finally, respondents who indicated that vaccination was a way for Western countries to get richer compared to those who did not believe so (aOR: 1.83; 95% CI: 1.46–2.30).

The following characteristics were most strongly associated with rejection of the COVID-19 vaccine: respondents who believed that vaccines never protected against severe disease, compared to those who did not (aOR:4.28; 95% CI: 2.71–6.77); respondents who had rejected a vaccine for themselves, or for a relative, in the past as compared to those who had not done so (aOR: 2.46; 95% CI: 1.73–3.48); respondents who had had a negative experience with vaccination in the past as compared to those who hat not (aOR: 1.84; 95% CI: 1.38–2.44); respondents who believed vaccines were always dangerous or cause diseases as compared to those that did not (aOR: 2.56; 95% CI: 1.70–3.86); respondents who believed vaccination to be a way to introduce diseases into Africa as compared to those who did not believed so (aOR: 3.97; 95% CI: 2.93–5.39); and finally, respondents who indicated that vaccination was a way for Western countries to make financial gain compared to those who did not perceive this to be true (aOR: 2.41, 95% CI: 1.83–3.18).

### Identification of respondent profiles associated with hesitancy to be vaccinated against COVID-19 in Chad

**Figure 3:**
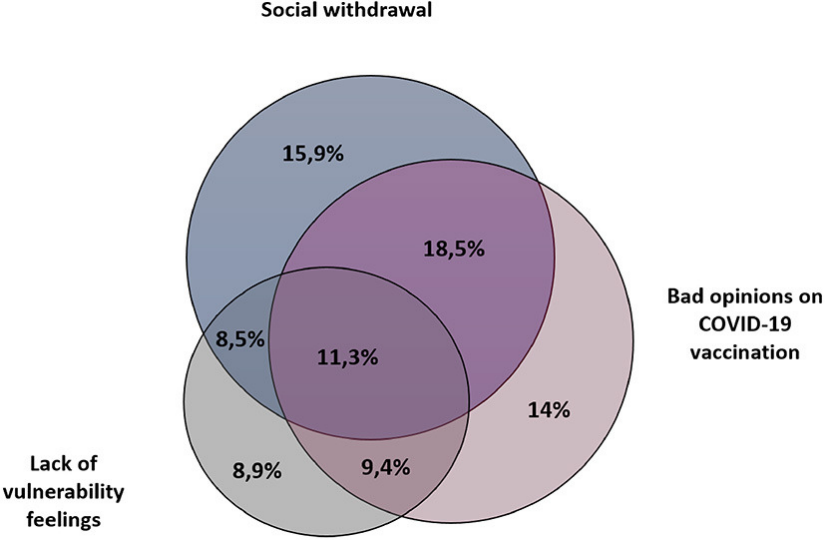
Multivariate data analysis overview. Venn diagram exhibiting groups with common factors in association with hesitancy to get vaccinated against COVID-19 in Chad. Three categories have been established: (1) Participants “with a bad opinion of the COVID-19 vaccination”; (2) participants with “lack of vulnerability feelings” and (3) participants considered to be in a “social withdrawal” situation.

We then sought to identify population profiles and factors in positive association with COVID-19 vaccination hesitancy using a multivariate analysis in a Venn diagram representation (Figure 3). We selected three factors that might be related to vaccine hesitancy, which allowed us to classify participants into categories associated with one or more of these hesitancy factors: participants “with a bad opinion of the COVID-19 vaccination”; participants with “lack of vulnerability feelings” and participants considered to be in a “social withdrawal” situation. We observe that for participants associated with hesitancy to COVID-19 vaccination, ~86% fall into at least one of these categories and ~39% into only one of these categories. The group with both a “poor opinion of COVID-19 vaccination” and “social withdrawal” represents the largest group with nearly 18%. Finally, only 13.5% of the participants who were hesitant to COVID-19 vaccination did not fit into any of these three categories and 11.3% fell into all three categories.

## Discussion

This study provides an assessment of the rate of intention to receive a COVID-19 vaccine in the general population of Chad, before the rollout of the vaccine, and describes the factors associated with vaccine hesitancy. Among 5,174, adult respondents, the percentage accepting COVID-19 vaccine were 47.9%. Compared to the overall acceptability rate of 63% reported in Africa (18) or the theoretical rate of 90% or more needed to achieve herd immunity, since the arrival of new, highly contagious variants of SARS-CoV-2 (24), results we have compiled in Chad are dramatically low. Moreover, uncertainty about reinfection after recovery from COVID-19, and relative disappointments about the duration of immunity provided by current vaccines, indicate that vaccination remains the most effective means to prevent severe forms of COVID-19 and overwhelming of health systems. In this study, we identified different factors related to socio-demographic characteristics, mistaken beliefs about vaccine, underestimation of the potential severity of the disease, that were associated with vaccine hesitancy.

In a study conducted in multiple African countries, acceptability of COVID-19 ranged from 33% in Cameroon to 84% in Liberia, with only the Democratic Republic of the Congo showing comparable rate (47%) to our study (18). Another recent study, conducted through telephone surveys, in six different countries from Sub-Saharan Africa, found an overall high level of willingness to be vaccinated against COVID-19, with rates ranging from nearly universal for Ethiopia (97.9%) to 60% for Mali (19). Nevertheless, surveys have often shown wide variations in acceptance rates of the COVID-19 vaccine for a given country, ranging for example between 31.4% (25) and 97.9% for Ethiopia (19). These large variations appear to be primarily due to temporal factors (26) which could be related to general public dissatisfaction with government responses to the pandemic and its economic consequences, or reflect personal experiences of COVID-19 illness or loss of life and livelihood, or be associated with changing perceptions of vaccine safety over time (27). In France, for instance, vaccine acceptance ranged from 62.0 to 77.1% in March/April and was only 58.9% in June 2021, likely related to concerns about the safety and efficacy of vaccines (10, 12).

Our study indicates that the acceptance rate of COVID-19 vaccination in Chad is one of the lowest in the Sub-Saharan zone among the countries surveyed, but also at a global level (9, 14, 18). The reluctance to vaccinate in Africa is generally related to a lack of knowledge that vaccines are the most effective public health interventions (6). Our data show that the main reasons given by hesitant respondents are lack of trust in COVID-19 vaccine, following by denial of the existence of COVID-19, beliefs in conspiracy theories of vaccines made to harm African people, and reliance in God for protection, while the most important reasons given by the respondents willing to take the COVID-19 vaccine is to protect themselves or their families. A comparable study found that 30% of Africans who reject the COVID-19 vaccine do so because they fear that the COVID-19 vaccine is being used as an excuse to “experiment” on Africans (18). It is very likely that the history of colonial medicine and the abuse of vaccine research in Africa diminishes confidence in current vaccines (6, 28). Mistrust of vaccines is certainly very difficult to combat, but confidence in vaccines is strongly connected with confidence in the organizations that provide them, and also in the origin of their development and production. The COVID-19 pandemic could serve as a wake-up call for Sub-Saharan Africa start to produce vaccines (29), which would most likely increase vaccine acceptance among many Africans.

Overall, COVID-19 vaccine hesitancy remains a highly prevalent problem in high-income countries. Individuals who were younger, females, not being of white ethnicity, and have a lower education or income levels, were more prone to vaccine hesitancy [15]. In this study, with regard to sociodemographic predictors of COVID-19 vaccine intention, our findings demonstrate that factors such as age, residence (urban or rural), education level, marital status and occupation could be significantly associated with COVID-19 vaccine undecidedness or rejection. However, unlike other studies (10), we did not find that reluctance to vaccinate was significantly associated with gender. Respondents with secondary or university education have lower odds of COVID-19 vaccine rejection. Our results also reflect a relationship between instability and precariousness of life status, whether professional, economical or matrimonial, and the intention to accept COVID-19 vaccination. Of particular significance in our study is the finding that rural respondents have significantly lower odds of undecidedness or rejection of COVID-19 vaccine as compared to urban respondents. In Sub Saharan Africa, rural residents generally have a significantly lower level of education. We can therefore assume that the reason for less hesitancy toward the COVID-19 vaccine in rural areas is not related the low level of education. The daily confrontation with infectious diseases, associated with an increasingly widespread practice of vaccination, and the low penetration of misconceptions on COVID-19 vaccine in rural areas may play a significant role in this outcome.

Concerning the characteristics related to personal situation, our data demonstrate that respondents with antecedents of COVID-19 signs and those with international travel plans have higher odds of rejection and undecidedness of COVID-19 vaccine. People who have experienced symptoms of COVID-19 may think that they are already immune and no longer need the vaccine, or that the disease is not fatal and therefore the vaccine is not useful. Indeed, 10% of the respondents hesitating to take the vaccine in our survey believe that COVID-19 would not kill them. On the other hand, personal characteristics such as having performed a COVID-19 diagnostic test or fear of contracting COVID-19 gave lower odds of undecidedness or rejection. Surveys conducted in France and Japan have shown a strong positive relationship between the fear of COVID-19 and the intention to get vaccinated (30, 31).

With regard to beliefs and attitudes, the main arguments against COVID-19 vaccination, with the strongest predictors in this category, were that vaccines never protect against dangerous diseases and that vaccination is a way of introducing diseases to Africa. Studies conducted in Japan and Puerto Rico, also indicated that people who had not been vaccinated against influenza were less likely to accept the COVID-19 vaccine (30, 32). The association between incorrect beliefs or beliefs in conspiracy theories have been well-documented in many studies across the world (7, 11, 18, 33). While vaccine hesitancy has a universal basis, some of the reasons may be more specific to some regions of Africa, related to local beliefs or traditional medicine. A survey conducted in N'Djamena between May and August 2020 on knowledge, attitudes and practices regarding COVID-19 showed that only 21.38% of the respondents had correct knowledge concerning COVID-19 (34). Our results indicate, 1 year later, that the gap between knowledge and ignorance, accuracy and false assumptions is still wide. Therefore, strategies for controlling COVID-19 hesitancy must vary and adapt based on these factors (35) requiring education strategies and health interventions that respond in a relevant way (36, 37). We believe that part of the solution could involve people who are trusted, such as health professionals, teachers, and religious leaders (38), but also by developing communication with local traditional practitioners.

Another finding of particular interest in our study is the proportion of “I don't know” responses in the category of attitudes and beliefs. These respondents are often significantly associated with vaccine undecidedness or rejection. For example, to the affirmation “vaccines are dangerous or cause diseases,” 23.9% of the respondents say “I don't know,” and their odds of undecidedness are higher as compared to those who say vaccines are never dangerous. The likelihood of these people answering 'I don't know' is always in favor of the incorrect attitude or belief. This may suggest that the “I don't know” response is given to hide a perceived politically incorrect response or position. However, a high rate of “not knowing” also indicates that lack of information is at least as important as misinformation. These results highlight the need to improve the dissemination of information on COVID-19 vaccination in the country. Our results are largely consistent with a recent review on general perceptions of COVID-19 vaccination in Africa, which found that factors driving vaccine hesitancy are fear of vaccine side effects, distrust of the pharmaceutical industry, and myths surrounding vaccination (14).

With the Figure 3, we intended to identify three categories of COVID-19 vaccine hesitancy determinants (Figure 3). Based on factors associated with vaccine hesitancy we considered a profile of participants “with a bad opinion of COVID-19 vaccination,” those who might feel “less vulnerable to severe forms of the disease” and the group that was considered to be in a “social withdrawal” situation that may be less concerned about spreading the SARS-CoV-2. We observed that almost 90% of participants with vaccine hesitancy fell into at least in one of these categories. About 40% of them fell only in one of these categories, indicating for these specific populations the possibility to target argumentative strategies to promote COVID-19 vaccination, such as countering misinformation, raising awareness of the potential seriousness of the disease, or rather approaches more orientated toward the integration of people who may feel marginalized. We also observed that 13% of participants were associated with all three patterns of resistance to COVID-19 vaccination, which will require additional attention and effort through the implementation of multiple strategies to encourage them to get vaccinated.

### Limitations

Our study has a number of limitations. First, the cross-sectional design adopted in the methodology does not allow for cause-and-effect inference.

Second, if the distribution of demographic characteristics of our study population was comparable to that of the country's population on many criteria (23, 39), our survey was conducted in only two different locations and we did not adjust the sampling method with respect to the place of residence. Our survey enrolled 78.5% urban respondents, compared to 21.5% rural respondents, while the geographic distribution of the population in Chad is 19% urban and 81% rural (6). Therefore, we cannot affirm that our study is representative of the national population. It is noteworthy that the results of our study show a sharp disparity in COVID-19 vaccine hesitancy depending on residence, with the odds of vaccine rejection being 10 times lower in rural respondents as compared to urban respondents. Therefore, the overall COVID-19 vaccine acceptance rate of our study might be underestimated. For logistical reasons, the rural population of Chad is the most difficult to vaccinate, which implies, in line with our results, that efforts in rural areas should focus on supply and storage difficulties, rather than on problems of reluctance to be vaccinated. Furthermore, our results are confirmed in the field, as we observe much lower opposition to COVID-19 vaccination in rural vs. urban areas.

Third, our study was conducted in 2021, over a year ago, just prior to the launch of the COVID-19 vaccination campaign, during a key period. Consequently, perceptions of COVID-19 vaccination may have evolved since.

Finally, we identify in this study three categories of determinants of COVID-19 vaccine hesitancy, allowing a first overview of the situation in Chad, but which will require an approach more in line with the official framework of WHO standards.

## Conclusion

This pilot study in Chad indicates an alarming level of acceptance of the COVID-19 vaccine in the general population, but also provides insight into which population communities are most likely to refuse vaccination and why. Our study highlights that vaccine hesitancy and refusal is higher in Chadian urban areas than in rural settings. In addition, we emphasize that COVID-19 vaccine hesitancy determinants are heterogeneous but may be grouped in three mains categories with bad opinion about vaccination, denial of COVID-19 severity, and relative social withdrawal. An awareness campaign to improve altruism and to debunk misconceptions about COVID-19 and vaccines, can increase vaccination intentions. We believe, that pro-vaccine messages at individual level should take into account the pattern of vaccine hesitancy. Factors that complicate vaccine acceptance in Africa should be further explored at country levels to improve the effectiveness of public health communication strategies.

## Data availability statement

The raw data supporting the conclusions of this article will be made available by the authors, without undue reservation.

## Ethics statement

Our study was approved by the Institutional Review Board of the University Hospital Complex le bon Samaritain (IRB N°016/CHU-BS/DG/2021-B). The patients/participants provided their written informed consent to participate in this study.

## Author contributions

GT, EM, OD, CG-V, FM, RL, MB, AC, ET, and LV were involved in the analysis and interpretation of the data. OD, GT, and FM did the drafting of the article. AC performed the statistical analysis. IR improved the quality of the English of the manuscript. FM wrote the final version all the manuscript. All authors revisited critically the manuscript for intellectual content, approved the version to be published and agree to be responsible for all aspects of the work, and involved in the conception and design of the study.
